# Differential spatial expression of peripheral olfactory neuron-derived BACE1 induces olfactory impairment by region-specific accumulation of *β*-amyloid oligomer

**DOI:** 10.1038/cddis.2017.349

**Published:** 2017-08-10

**Authors:** Seung-Jun Yoo, Ji-Hye Lee, So Yeun Kim, Gowoon Son, Jae Yeon Kim, Bongki Cho, Seong-Woon Yu, Keun-A Chang, Yoo-Hun Suh, Cheil Moon

**Affiliations:** 1 Department of Brain and Cognitive Sciences, Graduate School, Daegu Gyeungbuk Institute of Science and Technology, Daegu, Korea; 2 Convergence Research Advanced Centre for Olfaction, Daegu Gyeungbuk Institute of Science and Technology, Daegu, Korea; 3 Department of Pharmacology, School of Medicine, Gachon Medical School, Incheon, Korea

## Abstract

Olfactory dysfunction is a common symptom associated with neurodegenerative diseases including Alzheimer’s disease (AD). Although evidence exists to suggest that peripheral olfactory organs are involved in the olfactory dysfunction that accompanies AD pathology, the underlying mechanisms are not fully understood. As confirmed using behavioral tests, transgenic mice overexpressing a Swedish mutant form of human amyloid precursor proteins exhibited olfactory impairments prior to evidence of cognitive impairment. By measuring the expression of tyrosine hydroxylase, we observed that specific regions of the olfactory bulb (OB) in Tg2576 mice, specifically the ventral portion exhibited significant decreases in the number of dopaminergic neurons in the periglomerular regions from the early stage of AD. To confirm the direct linkage between these olfactory impairments and AD-related pathology, *β*-site amyloid precursor protein cleaving enzyme 1 (BACE1)—the initiating enzyme in A*β* genesis—and *β*-amyloid peptide (A*β*), hallmarks of AD were analyzed. We found that an increase in BACE1 expression coincided with an elevation of amyloid-*β* (A*β*) oligomers in the ventral region of OB. Moreover, olfactory epithelium (OE), in particular the ectoturbinate in which axons of olfactory sensory neurons (OSNs) have direct connections with the dendrites of mitral/tufted cells in the ventral part of OB, exhibited significant decreases in both thickness and cell number even at early stages. This result suggests that A*β* oligomer toxicity in the OE may have induced a decline in the number of OSNs and functional impairment of the olfactory system. We first demonstrated that disproportionate levels of regional damage in the peripheral olfactory system may be a specific symptom of AD with A*β* oligomer accumulation occurring prior to damage within the CNS. This regional damage in the olfactory system early in the progression of AD may be closely related to AD-related pathological abnormality and olfactory dysfunction found in AD patients.

Alzheimer’s disease (AD) is a neurodegenerative disorder characterized by memory decline and other functional cognitive impairments that result from the progressive degeneration of neurons in the brain.^1^ The AD brain has been histologically characterized by the presence of neuritic plaques and neurofibrillary tangles, which are composed of protein aggregates of amyloid *β* (A*β*) and tau, respectively.^2^ Specifically, soluble clusters of A*β* (A*β* oligomers) are involved in the early stage of AD prior to tau-mediated pathology.^3, 4^ The soluble monomeric forms, A*β*1–40 and A*β*1–42, are produced mainly in neurons from amyloid precursor protein (APP, ~120 kDa) via proteolytic process by *β*- and *γ*-secretases.^5, 6^ Although these monomeric forms of A*β* can drive formation of various oligomers in normal physiology, Aβ*56 (12-mer, ~56 kDa) and A*β*O (15-mer, ~80 kDa) have particularly high neurotoxicity.^7, 8^

Declining sensory functions are also common in neurodegenerative disorders including AD.^9^ A number of recent reports have revealed pathological events in the sensory organs of patients with AD.^10, 11^ Symptoms of olfactory dysfunction in particular were believed to be the result of neurodegeneration occurring in olfaction-related limbic cortices of the central nervous system (CNS) until the late 1980s.^12^ Although the precise mechanisms remain unclear, recent reports suggest that olfactory dysfunction appears in the early symptomatic stages of AD pathology^10, 13, 14^ and that sensory systems may be more vulnerable to AD pathology than the CNS as a whole. Nevertheless, the peripheral olfactory system in AD models has been not sufficiently investigated.

AD-related olfactory dysfunction has been explored using transgenic mice, Tg2576, expressing a Swedish mutant form of human amyloid precursor protein (APP) (KM670/671NL).^15, 16, 17, 18^ Tg2576 mice exhibit progressive olfactory impairment in an age-dependent manner correlated to stages of disease progression. In addition, 6-month-old, early stage of AD progression, Tg2576 mice exhibited impairment of olfactory habituation and discrimination resulting from deposition of A*β* in OB,^19^ implying that impairment of the central olfactory circuit is involved in olfactory dysfunction. Additionally, recent studies have shown that impairment of the peripheral olfactory system may also contribute to AD-related olfactory dysfunction. Overexpression of human APP in olfactory sensory neurons (OSNs) induces abnormal axonal projections of OSNs residing in the olfactory epithelium (OE) to the olfactory bulb (OB)^20^ and cell-autonomous cell death.^21^ Many previous studies have focused mainly on the OB and the central olfactory system rather than on OE and the peripheral olfactory organs, to explain the olfactory dysfunction observed in AD. Although some studies of olfactory behavior deficits and impaired OB functioning in this AD mouse model have been conducted,^22, 23^ there is little evidence to support the involvement of peripheral olfactory organs in olfactory dysfunction of the AD model.

In the current study, we first investigated the integrity of postsynaptic function and related structural changes between peripheral OE and OB for olfactory recognition using the Tg2576 mice at both early and late stages in order to understand the causes and mechanisms of the olfactory dysfunction related to AD. We also explored the feasibility of early AD diagnosis using the olfactory system.

## Results

### Tg2576 mice exhibit olfactory dysfunction before the onset of observable AD pathology

**Figure 1:**
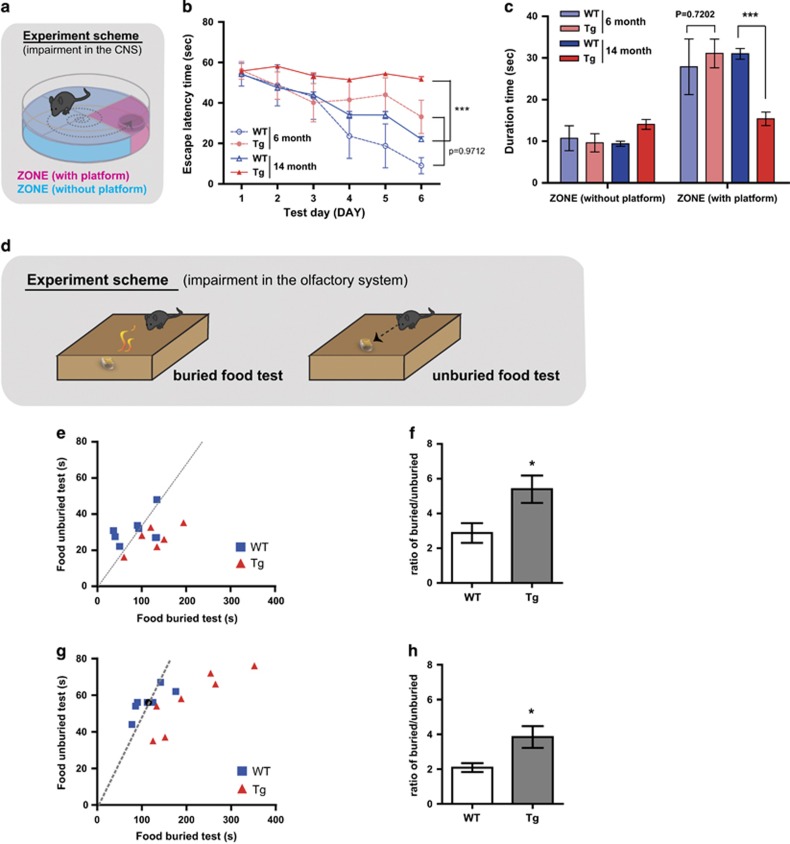
Olfactory impairment appears in 6-month-old Tg2576 mice without failure of spatial learning and memory. (**a**) The Morris water maze task was performed at 6 months (WT, *n*= 4; transgenic Tg2576 (Tg), *n*= 6) or 14 months of age (WT, *n* = 17; Tg, *n*= 16). (**b**) Training trials were conducted and the escape latency was measured for 6 days. ***P<0.001 by two-way ANOVA, using Prism software (GraphPad software). (**c**) The probe test was conducted 48 h after the final training session. A platform was located in zone with platform, and the durations that the mice of each group stayed in the zone without platform and the zone with platform were measured. ***P<0.001 by two way ANOVA, using Prism software (GraphPad software). (**d**) Illustration of scheme for buried/unburied food test. (**e** and **f**) Buried and unburied food tests in 6-month-old mice (WT, *n*=8; Tg, *n*=8). The latencies (**e**) to find buried or unburied food and their ratios (**f**) were measured (slope in the latencies graph (**e**) represents the average time to find buried or unburied food in WT mice) (**g** and **h**) Buried and unburied food tests in 14-month-old mice (WT, *n*=8; Tg, *n*=8). The latencies (**g**) to find buried or unburied food and their ratios (**h**) were measured (slope in the latencies graph (**g**) represents the average time to find buried or unburied food in WT mice) All data are presented as mean±S.E.M. Two-way ANOVA, using Prism software (GraphPad software), *P<0.05 and ***P<0.001 denote statistical significance

Because olfactory dysfunction has been observed in the early phase of AD pathology, we postulated that olfactory dysfunction may occur before the CNS defects in the Tg2576 mouse. First, using the Morris water maze test (Figure 1a), we found that 6-month-old Tg2576 mice exhibited slightly delayed escape latency during the 6-days training compared with wild-type (WT), while 14-month-old Tg2576 mice showed severe defects (Figure 1b). In addition, spatial learning, further assessed using the probe test, is impaired in the 14-month-old, but not the 6-month-old Tg2576 mice (Figure 1c). Next, we performed food-seeking tests to examine whether olfactory dysfunction appeared during the AD progression in Tg2576 mice. (Figure 1d). The olfactory dysfunction measured by the latency of seeking buried foods was aggravated between the 6 and 14 months in Tg2576 as well as WT mice. Interestingly, both 6- (Figures 1e and f) and 14- (Figures 1g and h) month-old Tg2576 mice exhibited a significant increase in the latency of seeking the buried food, but not the unburied food, compared with WT. These results demonstrate that olfactory dysfunction occurs even at the earliest stages of AD pathology. Collectively, we suggested that onset of olfactory dysfunction occurs early in AD pathology prior to the development of irretrievable impairments in the CNS of Tg2576 mice.

### Tg2576 mice experience a loss of dopaminergic periglomerular neurons in the specific glomeruli of ventral MOB

**Figure 2:**
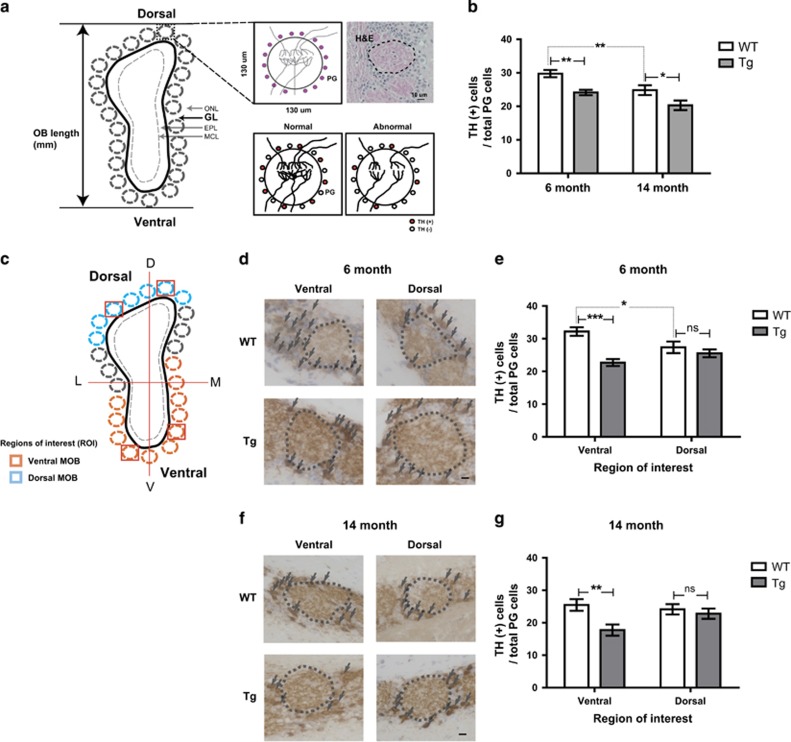
The number of dopaminergic periglomerular neurons (TH+PG cells) is decreased in Tg2576 mice compared with age-matched WT mice. (**a**) Depictions of coronal sections through the OB. Single glomerulus and H&E staining depicting the glomerulus with dopaminergic periglomerular neurons (TH (+) PG cells) are represented. Reduced activity between OSNs and mitral cells, or sensory deprivation, may result in profound decreases in TH expression. (**b**) Stereological analysis of TH (+) PG cells/total PG cells in the OB of WT *versus* Tg of 6- and 14-month-old. (**c**) Depictions of coronal sections through the OB with region of interest. Blue outline shading denotes the dorsal MOB. Orange outline shading denotes the ventral MOB. (**d** and **f**) Evolution of the TH (+) PG cells/total PG cells in WT *versus* Tg in 6-month-old (WT, *n*=7; Tg, *n*=7) (**d**) and 14-month-old (WT, *n*=9; Tg, *n*=8) (**f**). (**e** and **g**) Stereological analysis of TH+periglomerular cells/total periglomerular cells (WT control in each group) in 6-month-old (**e**) and 14-month-old (**f**) mice. All data are presented as mean±S.E.M. Scale bar=10 *μ*m. Two-way ANOVA, using Prism software (GraphPad software), *P<0.05, **P<0.01 and ***P<0.001 denote statistical significance

The glomerular layer (GL) layer mainly consists of periglomerular (PG) neurons located around the distinct glomeruli (Figure 2a). These PG neurons are well-known dopaminergic neurons expressing tyrosine hydroxylase (TH),^24^ and their survival is dependent on sensory input from OSNs.^25^ To examine whether the olfactory defect of Tg2576 mice could compromise the integrity of the OB, we measured the expression of TH in PG neurons (Figure 2a). The number of TH-positive PG neurons was significantly decreased in both 6- and 14-month-old Tg2576 mice (~20%) compared with age-matched WT mice (Figure 2b). Such declines in the number of TH-positive PG neurons were similarly observed in age-related decrements between 6- and 14-month-old WT mice (~20%), implying that Tg2576 mice may have a reduction of neural activity and integrity on the neuronal connection between the OE and OB^26^ and appeared level of decrements similar to the influences of age on olfaction.

The glomeruli are spatially, especially ventrally and dorsally, organized in GL of OB (Figure 2c). Notably, TH-positive PG neurons in 6-month-old Tg2576 mice significantly decreased in the specific glomeruli of ventral MOB, but not dorsal MOB (Figures 2d and e). These results were also observed in 14-month-old mice (Figures 2f and g). These results suggested that region-specific impairment of neural integrity in OB, especially ventral MOB, would be involved in the AD-related olfactory dysfunction, and this symptom would be observed in the early phase when AD pathology in the CNS has not yet begun.

### Tg2576 mice produce high levels of A*β* oligomer in the OB

**Figure 3:**
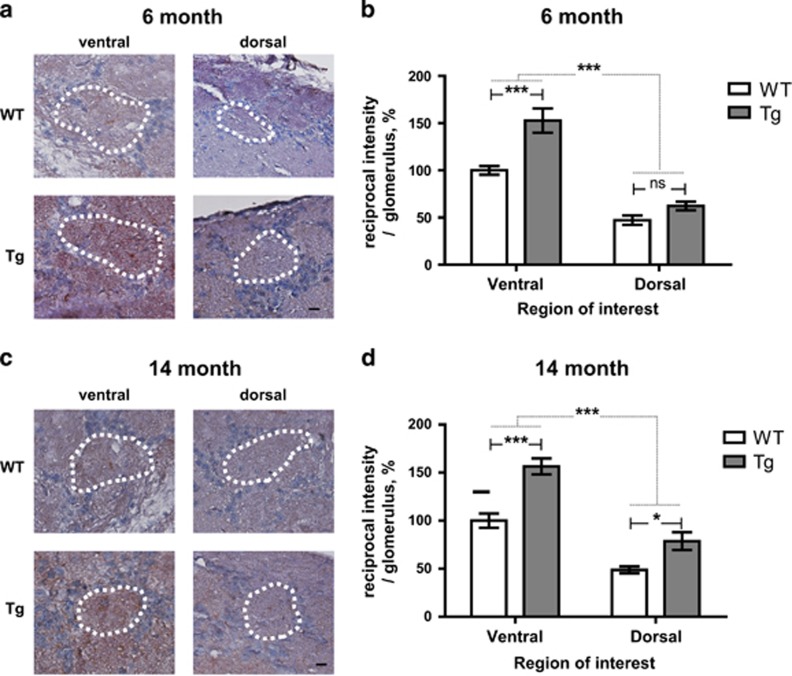
The intensity of the A*β* oligomer is increased in certain region OB of Tg2576 mice compared with age-matched WT mice. (**a** and **c**) Evolution of the A*β* oligomer in WT *versus* Tg in 6-month-old (WT, *n*=7; Tg, *n*=7) (**a**) and 14-month-old (WT, *n*=9; Tg, *n*=8) (**c**). (**b** and **d**) Stereological analysis of the A*β* oligomer intensity (WT control in each group) in 6-month-old (**b**) and 14-month-old (**d**) mice. All data are presented as mean±S.E.M. Scale bar=10 *μ*m. Two-way ANOVA, using Prism software (GraphPad software), *P<0.05 and ***P<0.001 denote statistical significance

From the previous results showing that soluble A*β* is able to disrupt network activity in neural circuits resulting in neural dysfunction, we confirmed A*β* oligomer immunoreactivity in the glomeruli of the OB of Tg2576 mice.^27^ Overall, we observed an overall increase in the A*β* oligomers expression in the OB of Tg2576 mice. Moreover, there were statistically significant differences between dorsal MOB and ventral MOB regardless of AD progression. Specifically, within the glomeruli of ventral MOB of 6-month-old Tg2567 mice, we observed increased levels of A*β* oligomer immunoreactivity compared with the dorsal MOB (ventral, ~53% dorsal, ~15%) (Figures 3a and b). In 14-month-old Tg2576 mice, glomeruli of ventral MOB also have highly accumulated immunoreactivity of A*β* oligomer and glomeruli of dorsal MOB also have significantly increased immunoreactivity (ventral, ~56% dorsal, ~30%) (Figures 3c and d). Collectively, we suggested that the accumulation of A*β* oligomers in specific regions may result in impaired neural integrity in specific glomeruli of ventral MOB of Tg2576 mice during AD progression.

### BACE1 is highly enriched in the axonal terminals of OSNs projecting to glomeruli and highly expressed in the ventral MOB in Tg2576 mice

**Figure 4:**
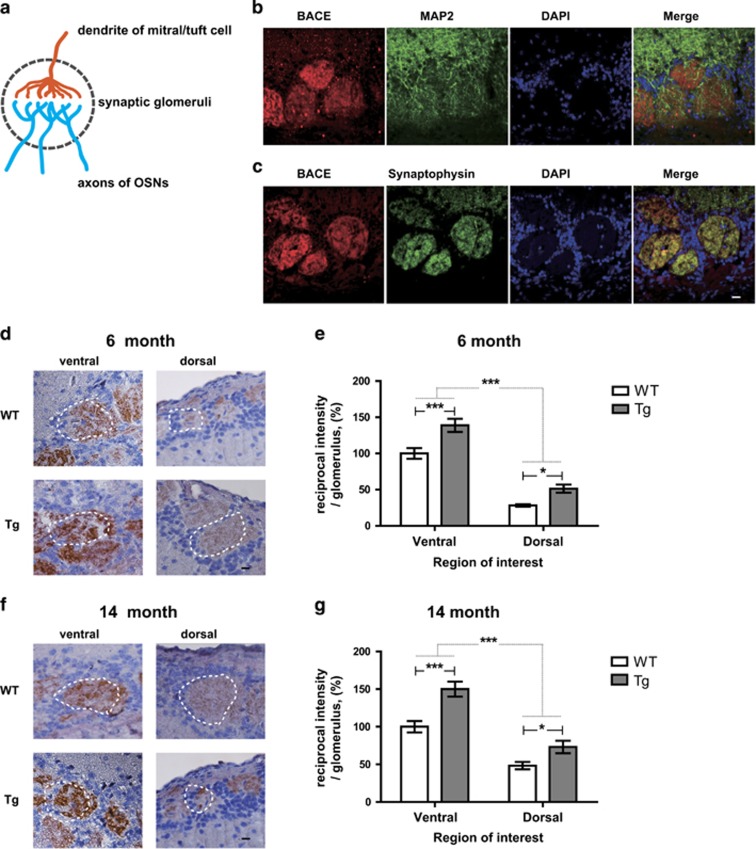
BACE1 is localized within presynaptic terminals and the intensity of the BACE1 is increased in certain region OB of Tg2576 mice compared with age-matched WT mice. (**a**) Illustration of coronal sections through the single glomerulus with presynaptic terminal of OSNs and postsynaptic region of mitral/tuft cells. (**b** and **c**) Representative images of coronal glomerulus sections from WT *versus* Tg in 6-month-old (Tg, *n*=4). Co-stained with BACE1 (red) and MAP2 (**b**) or synaptophysin (**c**) (green) antibodies and imaged by confocal microscopy. BACE1 immunoreactivity and synaptophysin signals significantly co-localize within OSN terminals, but BACE1 expression does not overlap with that of the somatodendritic marker MAP2 (green) within the glomerulus across all the condition while different signal intensity. Scale bar=10 *μ*m. (**d** and **f**) Evolution of the BACE1 in WT *versus* Tg in 6-month-old (WT, *n*=7; Tg, *n*=7) (**d**) and 14-month-old (WT, *n*=9; Tg, *n*=8) (**f**). (**e** and **g**) Stereological analysis of the BACE1 intensity (WT control in each group) in 6-month-old (**e**) and 14-month-old (**g**) mice. All data are presented as mean±S.E.M. Scale bar=10 *μ*m. Two-way ANOVA, using Prism software (GraphPad software), *P<0.05 and ***P<0.001 denote statistical significance

The *β*-site amyloid precursor protein cleaving enzyme 1 (BACE1) is a membrane-bound aspartyl protease abundant in the brains of AD patients and may be involved in the initial stage of A*β* generation. First, to investigate BACE1 localization in the olfactory system, we co-labeled BACE1 protein with a presynaptic terminal marker protein, synaptophysin, or a somatodendritic marker protein, microtubule-associated protein 2 (MAP2), in OB glomerulus (Figure 4a). Consistent with a previous report,^28^ we found that BACE1 immunoreactivity mainly co-localizes with synaptophysin in the glomeruli of MOB (Figure 4c); very little co-localizes with MAP2 (Figure 4b). These data indicate that BACE1 protein is highly expressed within axonal terminals of OSNs and other OB neurons in OB glomerulus rather than other somatodendritic postsynaptic neurons.

Based on previous studies and our own finding of A*β* oligomer expression in the OB, we postulated that BACE1 is involved in the regional accumulation of A*β* oligomers in ventral MOB. Thus, the expression of BACE1 in the OB glomeruli was assessed histologically. Expression of BACE1 was significantly enriched in the glomeruli of ventral MOB compared with the dorsal MOB both of 6- (ventral, ~39% dorsal, ~15%) (Figures 4d and e) and 14-month-old mice regardless of AD progression (ventral, ~42% dorsal, ~18%) (Figures 4f and g). It implies that the regional expression of BACE1 contributes to the accumulation of A*β* oligomers in the glomeruli of ventral MOB. Additionally, it significantly increased in all glomeruli of OB of both 6- (Figures 4a and b) and 14-month-old (Figures 4c and d) Tg2576 mice but still remains higher in the glomeruli of ventral MOB compared with the dorsal MOB (Figures 4e and g). These data indicate that increased expression of A*β* may reversibly enhance BACE1 expression in Tg2576 mice. Therefore, we suggest that region-specific expression of BACE1 leads to a vicious cycle of increased generation of A*β* oligomer and BACE1 expression in glomeruli of ventral MOB, finally contributing to the olfactory dysfunction. These data suggest that the BACE1 protein is enriched into presynaptic vesicles,^28^ thus we raised the possibility that peripheral OSNs are implicated with A*β* accumulation in the glomeruli of ventral regions of MOB leading to olfactory dysfunction in Tg2576 mice.

### The OE is impaired by cell death in the earlier phase of AD onset in Tg2576 mice

**Figure 5:**
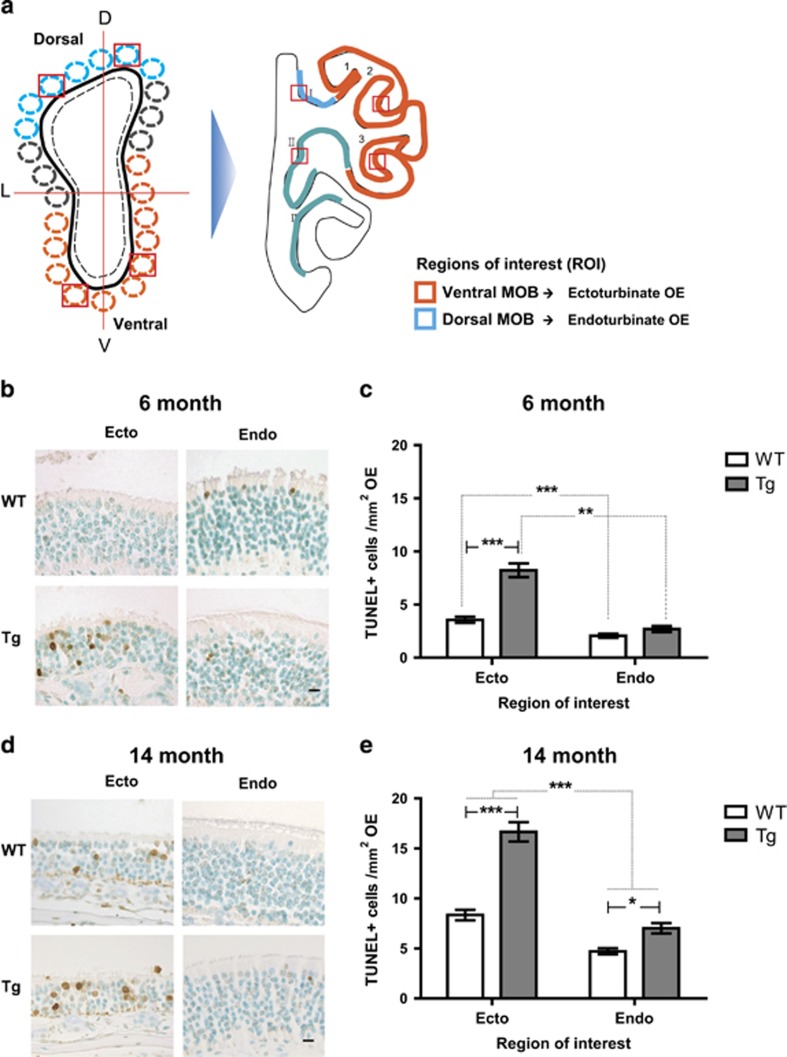
Specific region of OE shows massive cell death in Tg2576 mice. (**a**) Illustration of coronal sections through the OB and OE with region of interest. Depictions represent predetermined glomeruli in the OB and corresponding OE with region of interest. Blue outline shading denotes the dorsal MOB and orange outline shading denotes the ventral MOB. Blue outline shading denotes the endoturbinate OE and orange outline shading denotes the ectoturbinate OE. (**b** and **d**) Images of TUNEL staining (brown signal) in the ectoturbinate, and endoturbinate of the OE in (**b**) 6-month-old and (**d**) 14-month-old WT *versus* Tg mice. Methyl green was counterstained (sky blue signal). Scale bars represent 10 *μ*m. (**c** and **e**) Stereological analysis of the incidence of TUNEL-positive cells (TUNEL-positive cells/mm^2^ OE in each group) in the OE of 6-month-old (**c**) (WT, *n*=7; Tg, *n*=7) and 14-month-old mice (**e**) (WT, *n*=9; Tg; *n*=8). All data are presented as mean±S.E.M. Two-way ANOVA, using Prism software (GraphPad software), *P<0.05 and ***P<0.001 denote statistical significance

The rodent nasal cavity comprises a few regions formed by the bony turbinate extensions (turbinates) and molecular properties, and the OE is divided into ectoturbinals (1, 2, and 3) and endoturbinals (I, II, and III) according to anatomical locations exposed to air flows and odorants (Figure 5a). Additionally, depending on the OR expression, proliferation and relative maturation of the OSNs, OE is separated in four different ‘zones’, which correlate well with anatomical locations.^29^ OSNs in the OE have been shown to project their axons exclusively into predetermined glomeruli in the OB where their neural transmissions support the maintenance of neural circuits, which link the peripheral olfactory organ to the CNS.^30^ In addition, OSNs in the OE project to the MOB in a mutually exclusive pattern; the medial OE to the medial hemisphere of each MOB, the lateral OE to the lateral hemisphere, the central OE to the dorsal MOB, and the peripheral OE to the ventral MOB. Therefore, we analyzed histological characteristics in the regions of the OE including ectoturbinate and endoturbinate. The thickness of the OE was decreased in all regions to different extents (6 month: ecto ~10%, endo ~3% 14 month: ecto ~12%, endo ~8%), most notably in ectoturbinate of 6-month-old Tg2576 mice, while all regions in the 14-month-old mice were decreased (Table 1a). The number of cells in the OE also decreased in the ectoturbinate and the endoturbinate of 6-month-old Tg2576 mice and in all regions in 14-month-old mice (6 month: ecto ~11%, endo ~5% 14 month: ecto ~27%, endo ~14%) (Table 1b). Although Tg2576 mice displayed significant decreases in all regions tested (Tables 1a and b), both 6- and 14-month-old mice showed significant differences in both the thickness and the number of cells in a regional-dependent manner. These data indicate structural impairment of the OE in Tg2576 mice. In order to determine whether the impairment of the OE in Tg2576 mice was due to cell death, TUNEL staining was conducted. The number of TUNEL-positive cells increased significantly in ectoturbinate-specific regions of the OE in both 6- (Figures 5b and c) and 14-month-old Tg2576 mice (Figures 5d and e) compared with the endoturbinates of WT and Tg2576 mice. The most significant increase was observed in the ectoturbinate of both 6- and 14-month-old Tg2576 mice compared with WT. However, slight but significant increases were also observed in the endoturbinate of 14-month-old Tg2576 mice. Notably, almost all TUNEL-positive cells were observed in a layer in which OSNs reside (Figures 5b and d), indicating cell death of OSNs in Tg2576 mice.

### Tg2576 mice produce high levels of A*β* oligomers and induce cell death in OSNs layer of the OE

**Figure 6:**
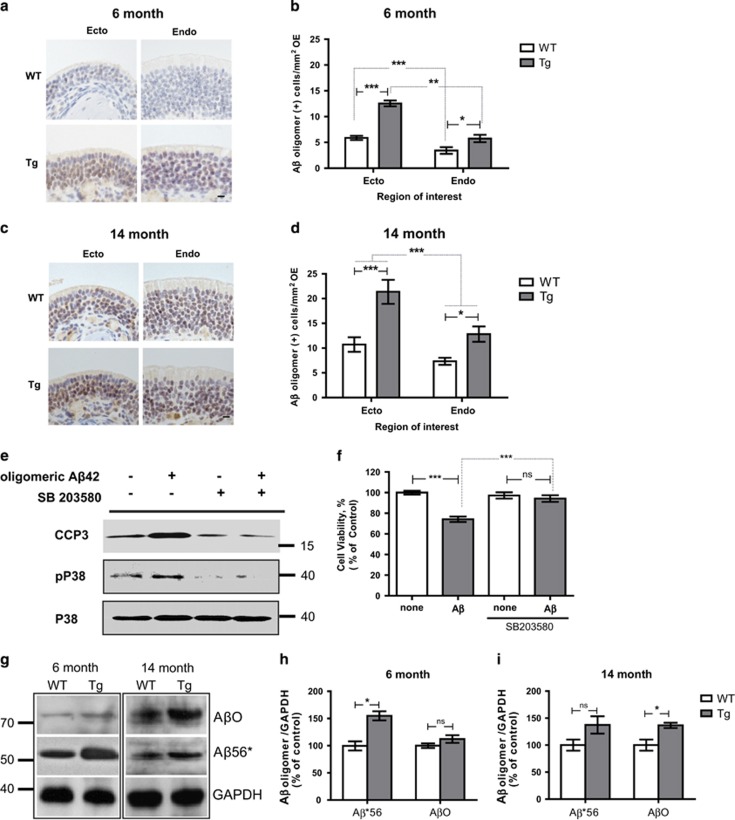
Expression of A*β* oligomer is significantly increased in the specific region OE of Tg2576 mice. (**a** and **c**) Immunohistochemistry of A*β* oligomers (brown signal) in the in the ectoturbinate, and endoturbinate of the OE of 6-month-old (**a**) and 14-month-old (**c**) WT *versus* Tg mice. Sections were lightly counterstained with H&E (violet signal). Scale bars=10 *μ*m. (**b** and **d**) Stereological analysis of the incidence (A*β* oligomer-positive cells/mm^2^ OE in each group) of cells exhibiting high immunoreactivity of A*β* oligomer in 6-month-old (**b**) (WT, *n*=7; Tg, *n*=7) and 14-month-old (**d**) (WT, *n*=9; Tg; *n*=8) mice. *P<0.05 and ***P<0.001 by one-way ANOVA, followed by Dunnett’s *post hoc* test. (**e**) Cultured OSNs were incubated with A*β* oligomer for 5 h in the presence or absence of SB 203580, and immunoblots of cleaved caspase-3 (CCP3), phosphorylated p38 (pP38), and p38 in OSNs treated with A*β* oligomers (25 *μ*M). (**f**) OSN viability was increased upon A*β* oligomer treatment. Inhibition of p38 significantly increased OSN survival under A*β* oligomer treatment. (**g**) Immunoblots of A*β*O (~80 kDa) and Aβ*56 (~56 kDa) in the OE of 6- or 14-month-old WT *versus* Tg mice. (**h** and **i**) Quantification of protein levels of A*β*O and Aβ*56 in 6-month-old (**h**) and 14-month-old (**i**) mice. Protein levels were normalized by reprobing the blots for GAPDH. The data were acquired from three independent experiments. All data are presented as mean±S.E.M. Two-way ANOVA, using Prism software (GraphPad software), *P<0.05 denote statistical significance

To elucidate whether the cell death of OSNs is correlated with generation of A*β* oligomers, we examined the abundance of A*β* oligomers in the OE of Tg2576 mice. Immunohistochemistry data revealed that immunoreactivity of A*β* oligomer was considerably stronger in ectoturbinates of the OE of both 6- (Figures 6a and b) and 14-month-old WT and Tg2576 mice (Figures 6c and d). Compared to WT mice, however, Tg2576 mice exhibit an overall upregulation of A*β* oligomer expression in the OE, in particular within the ectoturbinate. To examine if A*β* oligomers showed toxic effects to OSNs, we investigated the neurotoxicity induced by the A*β*1–42 peptide-derived diffusible ligands (ADDLs) *in vitro* using cultured OSNs.^31^ ADDLs induced OSN cell death with upregulated the cleavage of caspase-3, which is a final executor of apoptosis.^32^ In addition, phosphorylation of p38 MAPK, which is involved in the stress response^33^ (Figure 6e), was also increased, comparable to a previous report describing increased p38 MAPK activity in the AD human brain^34^ (Figure 6e). To elucidate whether the p38 signaling pathway was indeed involved in the ADDLs-mediated neurotoxicity, we co-treated OSNs with an inhibitor of p38 MAPK, SB 203580 at 20 *μ*M, with ADDLs. SB 203580 efficiently abolished OSN cell death as well as p38 activation (Figures 6e and f), indicating that A*β* oligomers may induce OSN cell death via p38 activation. Taken together, we suggest that the cell-autonomous death of OSNs by A*β* oligomers in peripheral organs, especially ectoturbinate of OE, of olfactory system, and it leads to impairment of integrity in glomeruli of OB, finally resulting in olfactory dysfunction in the earlier phase of AD pathology of Tg2576 mice.

Soluble monomeric forms, A*β*1–40 and A*β*1–42, are produced mainly in neurons from APP (~120 kDa),^5, 35^ and these monomeric A*β*s can drive the formation of various nonpathogenic oligomers as a part of normal physiological processes. Aβ*56 (12-mer, ~56 kDa) and A*β*O (15-mer, ~80 kDa) are highly neurotoxic without causing the formation of neuritic plaques or neurofibrillary tangles,^31^ and their high deposition have been found in the initial early stages of AD.^36^ By immunohistochemistry, we found that a toxic Aβ*56 was significantly increased in the OE of 6-month-old Tg2576 mice compared with WT, and was saturated in the OE of 14-month-old mice (Figure 6g). However, the other toxic A*β* oligomer, A*β*O (~80 kDa), was significantly increased in the OE of 14-month-old Tg2576 mice as compared with WT mice, but not in the OE of 6-month-old mice (Figures 6h and i). These data demonstrated that deregulated formation of A*β* oligomers, especially Aβ*56 rather than A*β*O, occurred in the ectoturbinate of Tg2576 mice and may be involved in cell death of OSNs resulting in olfactory dysfunction.

## Discussion

In the current study, we found olfactory dysfunction in 6-month-old Tg2576 mice before severe and irreversible defects in the CNS, including failure of spatial learning and memory. Comparably, a previous report has described that olfactory habituation and discrimination were significantly reduced in 6-month-old Tg2576 mice.^19, 37^ Considering that olfactory habituation and discrimination have been known to be secondary or tertiary olfactory sensory pathways mediated by the central olfactory circuit including synapses between the OB and the piriform cortex in CNS,^19^ defects of olfactory habituation and discrimination in Tg2576 mice result from impairment in the CNS. In contrast, our current results from anatomical analyses using immunohistochemistry and behavior analysis (i.e., food-seeking tests) provide new evidence that Tg2576 mice have defects in the primary olfactory circuit, specifically peripheral OE and glomerular layer of OB where the first synapses are formed, for odor recognition rather than central olfactory circuit.

Dopaminergic PG neurons in the GL of the OB have been shown to influence olfactory processing including odor detection and memory.^38, 39^ The maturation of PG neurons is supported by glutamatergic input from OSNs^40^ and is involved in the modulation of the excitability of mitral cells that form asymmetrical synapses with PG neurons.^41, 42^ Therefore, reduced activities between OSNs and mitral cells, or sensory deprivation may result in profound decreases in TH expression.^43, 44^ In the current study, glomerular regions in the ventral MOB were severely impaired in both 6-month-old and 14-month-old Tg2576 mice compared with the dorsal region, and this region exhibited enriched accumulation of A*β* oligomer with increased expression of BACE1. Our additional immunohistochemistry data revealed region-specific expression of BACE1 protein in glomeruli of ventral MOB, but not dorsal MOB. Previously, it has been demonstrated that presynaptic dystrophy by A*β* oligomers disrupts lysosome-dependent degradation of BACE1 protein.^28^ Based on our data and previous reports, we demonstrated that reduced neural activity from OSNs in OE to glomeruli of ventral OB increases BACE1 expression leading to enhancing accumulation of A*β* oligomers and a subsequently viscous cycle.

**Figure 7:**
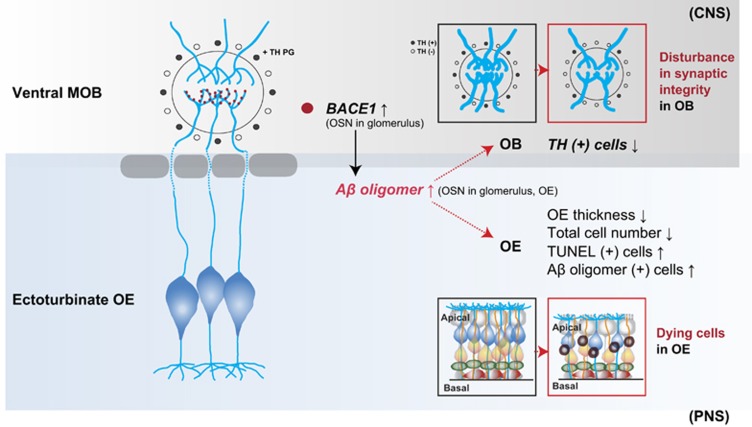
Working hypothesis of olfactory dysfunction and impairment in an AD mouse model. OE impairment and cell death occur in Tg2576 mice. In particular, the expression of A*β* oligomers is increased in the OE of Tg2576 mice compared with WT mice. In addition, the integrity of the connection between axons from the OE and dendrites in the OB is disturbed in Tg2576 mice compared with WT mice. Furthermore, dopaminergic periglomerular cells (TH+PG cells) are reduced in the glomerulus

One of our main question is, whether impairment of the PNS, but not the CNS, is involved in olfactory dysfunction in Tg2576 mice. Interestingly, our results showed that a specific group of OSNs, which are located in specific region of the OE, the ectoturbinate, and project their axons into glomeruli of ventral MOB, was severely degenerated in Tg2576 mice. Consistently, the region-specific deposition of A*β* oligomers in the ectoturbinate of Tg2576 (6- and 14-month old) mice was observed. This region-specific phenotype seems to result from region-specific expression of BACE1 (Figure 7). In addition, our biochemistry data revealed that Aβ*56 was upregulated in the OE during the early stage of AD in Tg2576 mice. It should be noted that Aβ*56 is one of the A*β* oligomer species correlated with cognitive deficits,^31^ and appears between the ages of 6 and 14 months in the Tg2576 mouse brain.^36^ Therefore, we suggest that evaluation of Aβ*56 deposition in the OE may be a potential-inducing factor of neural toxicity in early stage of AD. By contrast, A*β*O, an oligomer that induces direct cytotoxicity, significantly mediates cell death in the late stage of AD.^45, 46^ Taken together, olfactory dysfunction in the early stage of AD may be associated with alterations in the network activity by upregulated Aβ*56 synaptic toxicity. In contrast, olfactory dysfunction in the late stage of AD may be associated with massive cell death resulting from increased A*β*O cytotoxicity. That is, different types of oligomers may cause distinct types of deleterious effects in the olfactory system during the progress of AD.

Neuronal death and functional abnormality due to A*β* oligomers have been considered to be the main cause of AD.^1, 47^ OSNs are also cell-autonomously degenerated by OSN-specific expression of the Swedish mutated form of the human APP gene.^21^ In the current study, TUNEL-positive cells appeared to be almost entirely located in the OSN layer in Tg2576 mice, and *in vitro* cultured OSNs were cell-autonomously degenerated after treatment with the A*β* oligomer. Moreover, OSN-specific expression of the Swedish mutated form of the human APP gene disrupted the precise projections of their axons to the OB during synapse formation in 3-month-old Tg2576 mice without OSN death.^20^ Therefore, it is possible that abnormal axonal projections of OSNs are involved in the earlier olfactory dysfunction and in turn induce the cell death of OSNs with aberrant projections, resulting in more severe dysfunction in the early stage of AD.

An interesting finding from this study was that uneven regional damage could be the specific symptom of AD in the olfactory system. Comparably, the CNS also has such vulnerable regions to the AD such as default mode network regions of the AD,^48^ thus the olfactory system may also have susceptible region to AD-specific pathological processes. It is apparent from the data herein that there is a difference in the proliferation and maturation depending on the location of the OE,^49, 50^ suggesting the olfactory epithelium in different zones are differentially regulated by activity. Ectoturbinate and endoturbinate of OE belong to completely different regions and renewal of OSNs is not uniform.^51^ Interestingly, our results proposed that an AD-related protein has possible spatioselectivity in the olfactory system. Moreover, AD-specific pathological processes are highly concentrated in the damaged region of the olfactory system. Taken together, the mechanism of AD-mediated damage we proposed may be caused by problems of odorant detection in the OE and suggest that the PNS is critically impacted in early AD, ahead of damage to the CNS.

In summary, using a mouse model of AD, we observed early accumulation of *β*-amyloid in specific regions of the olfactory system including the OE. Specifically, BACE1 expression and cell death was highly correlated with a defect of *β*-amyloid positive presynaptic terminals of OB. Therefore, partial impairment of olfactory function in the progress of AD may show strong differences to other type of anosmic models. Therefore, particular odorants may show low detection tendency in a specific stage of AD progression because of region-specific degradation in the OE. Other reports showed the high sensitivity and specification of specific odorants in AD patients, for example, ‘peanut butter’ smell.^52^ Therefore, AD-specific symptoms in the olfactory system may present novel and feasible diagnostic targets for early diagnosis of AD.

## Materials and methods

### Transgenic mice

Tg2576 APP transgenic mice harboring the mutated human APP (695 amino acids) gene were obtained from Taconic (USA) and the production, genotyping, and background strain (C57BL/6 × SJL) have been described previously.^53^ All experiments were performed in accordance with ‘the Guidelines for Animal Experiments from the Ethics Committee at Seoul National University’ (IACUC No. SNU-091208-1).

### Morris water maze

To test spatial memory formation, the Morris water maze was performed as previously described with some modification.^54^ More specifically, the sizes of the circular water tank (diameter, 140 cm; height, 45 cm) and the platform (width, 13 cm; length, 13 cm; height, 17 cm) for the mice were modified. A platform was submerged and placed 0.5 cm below water made opaque using milk (depth, 17.5 cm) at the midpoint of one quadrant. Three training trials per day were conducted for 7 days, with a rotation order specified for each trial in a group. Mice were placed in the pool at one of the three possible quadrant starting positions (with the exception of the quadrant containing the platform). During each training trial, the time required to escape onto the hidden platform was recorded. Mice that found the platform were allowed to remain on it for 30 s, and were then returned to their home cages during the interval between trials. Mice that did not find the platform within 60 s were placed on the platform for 30 s at the end of each trial. The probe test was carried out for 48 h after the trials. For this test, the platform was removed from the pool and the trial was performed with a cutoff time of 60 s. The time spent in each quadrant was recorded.

### Food-seeking test

All behavioral and histological analysis was conducted by personnel blind to group inclusion. Food-seeking tests were performed at 6 and 14 months of age for the Tg2576 and WT groups (*n*=8 per group). The food-seeking test was modified slightly but otherwise performed as described previously.^55, 56^ Additionally, the unburied food-seeking test was also performed and compared to the buried food-seeking test under the same conditions to confirm olfactory dysfunction without cognitive impairment. For the buried food-seeking test, the speed with which an animal fasted for over 35 h could find a food pellet, either hidden underneath a layer of bedding or not, was measured. Therefore, this test was used to assess latency in finding food as both the buried pellet-seeking test and the unburied pellet-seeking test. Prior to the food-seeking tests, food restriction was applied for over 35 h to motivate animals to search for food. For the buried pellet-seeking test, mice were habituated in a clean home cage for 15 min prior to testing.^56^ A food pellet was buried ~2.5 cm under the bedding in a corner of the cage and a mouse was placed in the opposite corner. The time to first bite of the food pellet was measured using an installed digital camera (recording time: 15 min maximum based on the assumption that food-restricted mice which fail to use odor cues to locate the food within a 15-min period are likely to have deficits in olfactory abilities).

### A*β*42 oligomers preparation

A*β*42 oligomers were prepared as previously described.^57^ Briefly, the A*β*42 peptide (GL Biochem, Shanghai, China) was initially dissolved to a concentration of 1 mM in hexafluoroisopropanol. For its aggregation, the peptide was resuspended in dry dimethyl sulfoxide at 5 mM, and then added to Hams F-12 cell culture medium (PromoCell, Labclinics, Spain) to a final concentration of 100 mM at 4 °C for 24 h.

### Antibodies

The following commercially available antibodies were purchased: anti-A*β* oligomer (Millipore, Temecula, CA, #AB9234); anti-GAPDH (Millipore, #MAB374); anti-tyrosine hydroxylase (TH) (Millipore, # MAB318); anti-cleaved caspase-3 (Cell Signaling Technology, Beverly, MA, USA, #9661); anti-p38 (Cell Signaling Technology, #9212); anti-phosphorylated p38 (Cell Signaling Technology, #9211); GAPDH (Chemicon, Temecula, CA, USA, # MAB374).

### Western blot

OE was harvested in a prechilled lysis buffer (Sigma, St. Louis, MO, USA) containing a protease inhibitor cocktail (Roche, Branchburg, NJ, USA). Extracts were thawed, homogenized by sonication, and centrifuged at 10 000 rpm to remove cellular debris. Protein (100 *μ*g) from each sample was loaded for SDS-PAGE and transferred to 0.45 *μ*m PVDF membranes (Millipore). The membranes were blocked with 5% non-fat dry milk in Tris-buffered saline buffer with 0.1% Tween 20 and then incubated with primary antibodies. The primary antibodies used were anti-cleaved caspase-3 (1:1000), anti-A*β* oligomer (1:500), anti-p38 (1:1000), anti-phosphorylated p38 (1:1000), and GAPDH (1:1000). Immunoblots were visualized using a commercial development kit (Pierce, Rockford, IL, USA) and quantification was performed using the ImageJ program (NIH, Bethesda, MD, USA).

### Histology

Animals were anesthetized by intraperitoneal injection of 65 mg/kg ketamine with 5 mg/kg xylazine. The mice were then transcardially perfused with prechilled phosphate-buffered saline (PBS, pH 7.6). Heads were removed, skinned, and post-fixed overnight in 4% paraformaldehyde in PBS at 4 °C. The mandibles were discarded, and the trimmed heads were skinned and fixed by immersion in the same fixative for 1 week at 4 °C. The heads were decalcified in 10% EDTA (pH 7.0) for 1 week at 4 °C. After decalcification, the specimens were washed, dehydrated in increasing concentrations of ethanol, and transferred into xylene to clear the tissue. The specimens were infiltrated with paraplast and embedded. Frontal sections (coronal, 6 *μ*m) were cut serially from the tip of the nose to the posterior extension of the OE and OB, and each section was preserved on MAS-coated slides (Matsunami Glass Co., Tokyo, Japan).

We determined and measured the contents of the OE including thickness and cell number using hematoxylin-eosin (H&E) staining. For H&E staining, sections were deparaffinized and rehydrated in water. Samples were then stained with H&E, dehydrated again, mounted permanently with permount, and covered with coverslips.

### TUNEL staining assay

For TUNEL staining, deparaffinized and rehydrated sections were washed in PBS for 5 min and treated with proteinase K (10 *μ*g/ml) in PBS at room temperature for 30 min. After washing in distilled water for 5 min, the TUNEL incubation solution (Millipore) containing TdT buffer, cobalt chloride, TdT, and biotin-16 dUTP was prepared in accordance with the manufacturer’s protocol. The sections were incubated in TdT buffer for 1 h at 37 °C and then exposed to a stop solution for 10 min. After washing twice in PBS (5 min each), the sections were incubated with anti-digoxigenin peroxidase conjugate at room temperature for 30 min. Finally, sections were incubated in the DAB solution for several minutes. The fragmented DNAs were visualized as a brownish color inside the nuclei. The sections were counter-stained with methyl green before being dehydrated and cleared through graded alcohols and xylenes.

### Immunohistochemistry

For immunohistochemistry, the endogenous peroxidase in the samples was quenched using 3% hydrogen peroxide in 10% methanol for 30 min. In order to retrieve antigenicity, the samples were boiled in 0.1 M citrate-buffered saline (pH 6.0) for 5 min. The sections were allowed to cool for 30 min, and were then washed twice in PBS (5 min each). After washing in PBS-T (0.1% Triton X-100 in PBS) for 30 min, the sections were blocked for 1 h in blocking solution (5% normal donkey serum and 1% BSA in PBS-T) and incubated with primary antibodies overnight at 4 °C. Anti-A*β* oligomer (1:200), anti-A*β* (1:500), and anti-TH (1:200) antibodies were used. After washing in PBS-T, the sections were incubated with a biotinylated secondary antibody for 1 h at room temperature. Subsequently, sections were treated with the avidin-biotin-peroxidase complex (Vectastain Elite ABC kit) for 1 h at room temperature. The sections were developed for 5 min in a 0.05% DAB solution, and were counter-stained with hematoxylin. Images were captured with a Nikon digital camera (DS-Ri1) attached to a Nikon-Eclipse-90i microscope (Nikon Corp., Tokyo, Japan).

### Imaging and data analysis

All images were captured using a Nikon ECLIPSE 90i microscope and a Nikon DS-Ri 1 digital camera (Nikon Inc). Digital images were processed using Adobe Photoshop, adjusting only brightness, contrast, and color balance. The numbers of immunoreactive cells were counted manually by two independent investigators blinded to the experimental conditions. The stained OEs were divided into eight regions according to the same orientation. Three slides for each of the 10 regions were used for histological measurements or immunohistochemistry. The OB was divided into eight regions according to the same orientation. Three slides for each animal were analyzed and observed under a microscope (× 400). OE thickness was measured from the basal lamina to the apex by examining the structure of the hematoxylin-stained olfactory mucosa. Subsequently, the number of stained nuclei was counted in an area of 2500 *μ*m^2^ in the OE and for a glomerulus in the OB. In order to address this fixed area on the slide, the septum length in the OE and the OB length were measured. Immunoreactive cells were counted from the endoturbinate (2 × 2 regions), and the ectoturbinate (2 × 2 regions) in the OE and were counted from the glomerulus (2 × 2 regions) in the OB from three tissue sections per animal. To quantify the reciprocal intensity, by measuring the intensity per unit area, with ImageJ, using the color deconvolution plug-in (http://wiki.imagej.net/Colour_Deconvolution).^58, 59^ The target unit area of images processed using the color deconvolution tool in imageJ to separate brown from other color in images. The area of brown staining was then quantified and divided by the total area to yield a percentage of staining area. Stereological analyses were conducted using Prism software (GraphPad Software, Inc., La Jolla, USA). Comparisons between WT and Tg2576 mice were conducted using two-way ANOVAs. Results are presented as mean±S.E.M. *P*-values of ≤0.05 were considered to be statistically significant.

### Primary culture of OSNs

Cultures were prepared as previously described with some modifications.^60^ OSNs which were obtained from Sprague-Dawley rats were plated at a density of 2 × 106 cells/ml on tissue culture dishes (Falcon, Lincoln Park, NJ, USA) coated with 25 *μ*g/ml laminin (BD Bioscience, San Diego, CA, USA) in modified Eagle’s medium containing d-valine (MDV, Welgen Inc., Worcester, MA, USA). Cultures are placed in humidified 37 °C incubator receiving 5% CO_2_. On 2 days *in vitro* every day thereafter, cells are fed with MDV containing 15% dialyzed fetal bovine serum (Gibco, Rockville, MD, USA), gentamicin, kanamycin, and 2.5 ng/ml nerve growth factor. Two days prior to use, the culture medium is changed to medium without nerve growth factor.

## Figures and Tables

**Table 1:** Thickness and cell numbers of the OE were decreased in Tg2576 mice compared with age-matched WT mice

**(a)**	**OE thickness, μm**			
	**6 months**		**14 months**	
	**WT**	**Tg**	**WT**	**Tg**
Ecto	47.2±3.2	43.4±3.6 (**)	48.6±8.3	42.8±4.6 (***)
Endo	76.1±6.7	73.9±9.3 (*)	77.1±8.1	70.2±3.2 (***)

**(b)**	**Total number of cells (2.5 mm**^**2**^)			
	**6 months**		**14 months**	
	**WT**	**Tg**	**WT**	**Tg**
Ecto	42.7±5.6	38.0±5.7 (**)	41.4±3.0	30.1±2.9 (***)
Endo	83.1±7.0	79.0±8.8 (*)	61.0±10.3	52.1±9.9 (***)

(a) Thickness in each region of the OE in 6-month-old WT (*n*=7) *versus* Tg (*n*=7) and 14-month-old WT (*n*=9) *versus* Tg (*n*=8). Two-way ANOVA, using Prism software (GraphPad software), *P<0.05, **P<0.01, and ***P<0.001 denote statistical significance.

(b) The number of cells in each region of the OE in 6-month-old WT (*n*=7) *versus* Tg (*n*=7) and 14-month-old WT (*n*=9) *versus* Tg (*n*=8). Two-way ANOVA, using Prism software (GraphPad software), *P<0.05, **P<0.01, and ***P<0.001 denote statistical significance.
